# Skeletal Muscle Involvement Pattern of Hereditary Transthyretin Amyloidosis: A Study Based on Muscle MRI

**DOI:** 10.3389/fneur.2022.851190

**Published:** 2022-05-02

**Authors:** Xujun Chu, Kang Du, Yuwei Tang, Xutong Zhao, Meng Yu, Yiming Zheng, Jianwen Deng, He Lv, Wei Zhang, Zhaoxia Wang, Yun Yuan, Lingchao Meng

**Affiliations:** ^1^Department of Neurology, Peking University First Hospital, Beijing, China; ^2^Beijing Key Laboratory of Neurovascular Disease Discovery, Beijing, China

**Keywords:** amyloidosis, transthyretin, MRI, fatty infiltration, polyneuropathy

## Abstract

**Objects:**

This study was intended to explore the characteristics of muscle magnetic resonance imaging (MRI) of patients with hereditary transthyretin amyloidosis (ATTRv amyloidosis) prospectively.

**Methods:**

The clinical data of 20 patients with ATTRv amyloidosis at our hospital between July 2020 and August 2021 were analyzed. MRI of lower limbs including calf muscles was performed in all these 20 patients and MRI of thigh muscles was performed in 16 of them.

**Results:**

The mean age of the 20 patients with ATTRv amyloidosis was 44.2 years (ranging from 26 to 60) whose mean duration of weakness was 23.3 ± 23.0 (ranging from 0 to 84) months. All the patients presented with polyneuropathy, and 18 of them with weakness in their lower limbs. Muscle involvement was selective in these patients with ATTRv amyloidosis. The posterior group of muscles was heavily fatty, and the soleus muscle was the most heavily involved. The proportion of fatty infiltration scores at the calf level was higher than at the thigh level with paired comparison for most patients. Three of these patients had more severely fatty infiltration of muscles at the thigh level. The fatty infiltration of posterior compartments at the calf level was highly consistent with neuropathy impairment scores of lower limbs (weakness), the strength of ankle plantar flexion muscles, and the amplitude of the compound muscle action potential of the tibial nerve.

**Conclusions:**

It was found that the pattern of muscle fatty infiltration was consistent with a distal-to-proximal gradient on the whole and that proximal involvements in MRI of lower limbs in some patients could also be observed. Selective fatty infiltration of muscles of posterior compartments and fatty infiltration of the soleus muscle might be typical of ATTRv amyloidosis.

## Introduction

Hereditary transthyretin amyloidosis (ATTRv amyloidosis) is an autosomal dominant disorder caused by *TTR* gene mutation and characterized by extracellular deposition of TTR amyloid fibrils in the peripheral nerve, heart, and other organs, leading to sensory-motor axonal polyneuropathy, cardiomyopathy, autonomic dysfunction, nephrotic syndrome, and vitreous opacities (1, 2). ATTRv amyloidosis is considered a progressive and fatal disease with early-onset (<50 years) and late-onset (≥50 years) conditions. Electrophysiological studies generally reveal sensory-motor axonal polyneuropathy, sometimes with demyelinating features (2, 3). Pathologically, the disease is characterized by the loss of nerve fibers with TTR deposition. The loss of large myelinated fibers tends to be conspicuous in late-onset cases. Vessels destroyed by TTR accumulation may participate in the pathogenesis of neuropathy (4, 5). Some patients with ATTRv amyloidosis showed a myopathic phenotype characterized by proximal weakness. Needle electromyography (EMG) revealed short-duration, low-amplitude motor unit action potentials with early recruitment. Perivascular and perimysium amyloid deposition reportedly occurred in patients with ATTRv amyloidosis by muscle biopsy, suggesting that myopathy deserves attention in ATTRv amyloidosis (6–8).

Muscle magnetic resonance imaging (MRI) is a painless and rapid tool to assess the pattern of muscle involvement in neuromuscular disorders. In hereditary neuropathies, muscle MRI can help determine the level of denervation by documenting the pattern of muscle atrophy and fatty infiltration. The length-dependent denervations were confirmed in many hereditary neuropathies, and muscle MRI exhibited a distal-to-proximal gradient of muscle damage (9, 10). Muscle MRI was also applied to amyloidosis. The reticular short-tau inversion recovery (STIR) hyperintensity and hypertrophy of adductor magnus have been described in systemic amyloidosis (11–13). This study aimed to explore the features of muscle MRI in ATTRv amyloidosis prospectively.

## Materials and Methods

### Patients and Clinical Evaluations

Twenty patients who had been diagnosed with ATTRv amyloidosis between July 2020 and August 2021 at Peking University First Hospital based on clinical manifestations and molecular analysis of the *TTR* gene were enrolled. All these patients with ATTRv amyloidosis had their disease history recorded in detail and underwent a focused neurological examination of measurement scales, including weakness scores of Neuropathy Impairment Score-Lower Limbs (NIS-LL), which assessed motor functions in lower limbs, and strength of ankle plantar flexion (dominated by gastrocnemius and soleus muscle) and ankle dorsiflexion (dominated by the tibialis anterior muscle) in NIS-LL weakness scores (a total value of bilateral legs). The NIS-LL contains sensation, strength, and reflexes of lower limbs with scores ranging from 0 to 88, we used the NIS-LL weakness scores including the strength of lower limbs with scores ranging from 0 to 64. The strength of ankle plantar flexion muscles and ankle dorsiflexion muscles in NIS-LL weakness scores ranged from 0 to 8, respectively. Nerve conduction studies (NCSs) and needle EMG were performed in these patients with ATTRv amyloidosis according to the standard protocol. The compound muscle action potential (CMAP) of the bilateral tibial nerves and common peroneal nerves of each of these patients were included in this study, and the amplitude of the CMAP was defined as the mean value of bilateral nerves. The severity of disability was evaluated according to the Coutinho stages of ATTRv amyloidosis (stage 0, no symptoms; stage I, unimpaired ambulation, mostly with mild sensory, motor, and autonomic neuropathy in the lower limbs; stage II, assistance for ambulation required, mostly with a moderate motor, sensory, and autonomic impairment of the four limbs; and stage III, wheelchair-bound or bedridden status with severe sensory, motor, and autonomic involvement of all limbs).

This study was conducted as recommended by the ethics committee of Peking University First Hospital with written informed consent from all patients.

### Muscle MRI Scans and Interpretation

Muscle MRI of nine patients was conducted in 3.0 T MR scanners (Philips Healthcare, Best, the Netherlands), with the following sequences: axial T1-weighted spin-echo series with 400/15 (repetition time, ms/echo time, ms). Muscle MRI of 11 patients was conducted in 1.5 T MR scanners (GE Healthcare, Waukesha, WI, USA), with the following sequences: axial T1-weighted spin-echo series with 450/12 (repetition time, ms/echo time, ms). Conventional axial T1-weighted spin-echo series (T1WI) of the calf muscles was obtained in all these 20 patients according to standard protocols (14), while that of the thigh muscles was obtained in 16 of these patients. All scans were independently interpreted by an experienced radiologist and a neurologist, who was blinded to the clinical information and molecular diagnosis during image review. The extent of fatty infiltration of individual muscles was graded on axial T1WI using a modified 0–5 Mercuri's point scale (15) as follows: stage 0, normal muscle appearance (score 0); stage 1, occasional scattered areas of increased density (score 1); stage 2a, numerous discrete areas of increased density <30% of the individual muscle volume (score 2); stage 2b, increased areas of confluent density, 30–60% of the individual muscle volume (score 3); stage 3, washed-out appearance due to increased areas of confluent density, more than 60% of the individual muscle volume (score 4); and stage 4, end-stage appearance, muscle entirely replaced by areas of confluent density (score 5). The Mercuri's point scale was used to measure and compare the fatty infiltration (bilateral mean values) in muscles.

A total of 28 muscle groups were selected, 16 at the thigh level (gluteus maximus (GM), obturator internus (OI), obturator externus (OE), rectus femoris (RF), vastus lateralis (VL), vastus medialis (VM), vastus intermedius (VI), sartorius (SA), adductor magnus (AM), adductor longus (AL), adductor brevis (AB), gracilis (GR), semitendinosus (ST), semimembranosus (SM), long head of biceps femoris (BFL), and short head of biceps femoris (BFS) and 12 at the calf level (tibialis anterior (TA), extensor hallucis longus (EH), extensor digitorum longus (ED), peroneus brevis (PB), peroneus longus (PL), popliteus (PO), tibialis posterior (TP), flexor digitorum longus (FD), flexor hallucis longus (FH), soleus (SO), medial head of gastrocnemius (MG), and lateral head of gastrocnemius (LG). The thigh muscles were divided into (1) the anterior compartments: SA, RF, VI, VL, and VM; (2) the medial compartments: GR, AM, AL, and AB; and (3) the posterior compartments: BFL, BFS, SM, and ST. The calf muscles were divided into (1) anterior compartments: TA, EH, and ED; (2) lateral compartments: PB and PL; and (3) the posterior compartments: MG, LG, SO, PO, TP, FD, and FH.

The average fatty infiltration scores of anterior, posterior, and lateral compartments were calculated and the proportion of fatty infiltration at both calf and thigh levels was expressed as follows: the proportion of fatty infiltration = total fatty infiltration scores (patient)/ [5 × (16 or 12)] × 100%.

### Statistical Analysis

IBM SPSS Statistics, version 24, was used for statistical analysis. The clinical data on the patients were included for descriptive statistics. Variables were presented as the mean standard deviation. Continuous variables between two defined groups were compared using a paired nonparametric test (Mann–Whitney test), while categorical variables were compared using the chi-square test or Fisher's exact test where appropriate. Two-sided *p* values were calculated for all analyses, and *p* < 0.05 was considered statistically significant. Spearman analysis was used to test the correlation between MRI parameters and the clinical data.

## Results

### Clinical Features of the ATTRv Amyloidosis Patients

The mean age of patients with ATTRv amyloidosis was 44.2 (ranging from 26 to 60) years. The mean duration of weakness was 23.3 ± 23.0 (ranging from 0 to 84) months. In clinical staging, 11 (55.0%) of these patients were assigned to Coutinho stage I, 8 (40.0%) to Coutinho stage II and 1 (5.0%) to Coutinho stage III. Eleven of these patients with ATTRv amyloidosis started with paresthesia of the lower limbs, followed by other onset symptoms such as blurred vision in four patients, alternating diarrhea and constipation in two patients, sexual dysfunction, orthostatic hypotension, and carpal tunnel syndrome in one patient, respectively. All these patients presented with polyneuropathy when enrolled in this study, and 18 (90.0%) of them manifested weakness of the lower limbs, namely prominent distal, prominent proximal, and no weakness in sixteen, two, and two patients, respectively. Thirteen (65.0%) of them were early-onset (<50 years). *TTR* gene screening was performed, with Val30Met mutation in five patients, and Gly83Arg, Val30Leu mutation in three patients, respectively. Lys35Asn, Ala97Ser, Val30Ala, Glu42Gly, Ala36Pro, Phe33Val, Gly47Arg, Ser77Phe, and Asp38Val mutation was in one patient, respectively (Table 1). The mean NIS-LL (weakness) was 27.2 ± 24.4, the mean NIS-LL (weakness) of the strength of ankle dorsiflexion muscles was 1.6 ± 1.5, and the mean NIS-LL (weakness) of the strength of ankle plantar flexion muscles was 1.4 ± 1.5. Nerve conduction studies were also performed in all these patients with predominant axonal impairment and needle EMG showed neurogenic damage in 15 out of all the patients. The mean value of the amplitude of the CMAP of the tibial nerve was 1.5 ± 2.3 mV, and the mean value of the amplitude of the CMAP of the common peroneal nerve was 1.4 ± 1.9 mV.

**Table 1:** Demographic and clinical characteristics of patients with ATTRv amyloidosis.

**Patient No**.	**Age at onset (y.)/ Gender**	**Disease duration of weakness (mo.)**	**Coutinho stage**	**Mutation**	**LL weakness pattern**	**Phenotype**	**NIS-LL (weakness)**	**Strength of the tibialis anterior muscle in NIS-LL (weakness)**	**Strength of gastrocnemius and soleus muscles in NIS-LL (weakness)**	**The amplitude of the CMAP of the tibial nerve (mV)**	**The amplitude of the CMAP of the common peroneal nerve (mV)**	**Needle EMG**
1	38/M	72	II	Lys35Asn	distal>proximal	PN+AN+C+E	41	3.25	3.25	0	0.193	NA
2	43/M	36	II	Val30Met	distal>proximal	PN+C+AN+H	37.5	3.75	3.25	0	1.505	Neurogenic
3	42/M	12	I	Gly83Arg	distal>proximal	PN+E+AN+C+CTS	22	3.75	3.5	0.39	0.215	Neurogenic
4	59/M	24	I	Ser77Phe	distal>proximal	PN+C+AN+E	17	1.5	1.5	0.76	1.145	NA
5	33/M	0	I	Ala36Pro	no weakness	PN+AN+C+CTS	0	0	0	10.05	1.595	Neurogenic
6	27/M	12	I	Glu42Gly	distal>proximal	PN+AN+C+Cough	30.5	1	1	1.4	1.4	Neurogenic
7	56/M	36	II	Val30Met	distal>proximal	PN+AN+C+Cough	20.75	3.125	1	1.38	0	Neurogenic
8	48/F	0	I	Val30Leu	no weakness	PN+AN+C	0	0	0	4.3	1.225	Neurogenic
9	60/M	84	III	Val30Met	distal>proximal	PN+AN+C	38	4	4	NR	0	Neurogenic
10	58/M	12	I	Val30Met	proximal>distal	PN+AN+C	4	0	0	1.415	1.52	NA
11	27/F	6	I	Gly47Arg	distal>proximal	PN+AN+C+E	6	0.5	0	0.115	0.325	Neurogenic
12	26/M	0	I	Phe33Val	distal>proximal	PN+C+AN+E+Cough	2	0	0	0.485	0.71	Neurogenic
13	55/F	12	II	Val30Met	distal>proximal	PN+AN+C+E+Cough+H	21	3	3	2.8	0.655	Neurogenic
14	27/F	12	I	Val30Ala	distal>proximal	AN+PN+C+Cough	12	0	0	1.725	4.28	NA
15	42/M	12	I	Gly83Arg	distal>proximal	PN+AN+C+E	12	1	1	3.35	5.6	Neurogenic
16	43/M	16	I	Gly83Arg	distal>proximal	PN+AN+E	20	2.5	2.5	0	0	Neurogenic
17	50/F	24	II	Val30Leu	proximal>distal	PN+AN+C+Cough	42.5	2.5	2.5	0	0	Neurogenic
18	57/M	12	II	Asp38Val	distal>proximal	PN+AN+C	11	1.5	0	0.5	6.325	NA
19	47/M	48	II	Ala97Ser	distal>proximal	PN+AN+C	20	1	1	0.445	0.45	Neurogenic
20	45/M	36	II	Val30Leu	distal>proximal	PN+AN+C	46.5	3.875	3.875	0.75	0.2	Neurogenic

*AN, autonomic neuropathy; ATTRv amyloidosis, hereditary transthyretin amyloidosis; C, cardiopathy; CMAP, compound muscle action potential (mean values of bilateral nerves); CTS, carpal tunnel syndrome; E, eye; EMG, electromyography; F, female; H, hearing loss; LL, lower limbs; M, male; mo., months; NA, not available; NIS-LL (weakness): neuropathy impairment score of lower limbs weakness; PN, peripheral neurology*.

*Strength of ankle dorsiflexion muscles was dominated by tibialis anterior muscle; Strength of ankle plantar flexion muscles was dominated by gastrocnemius and soleus muscles; y, years*.

### MRI Findings

**Figure 1:**
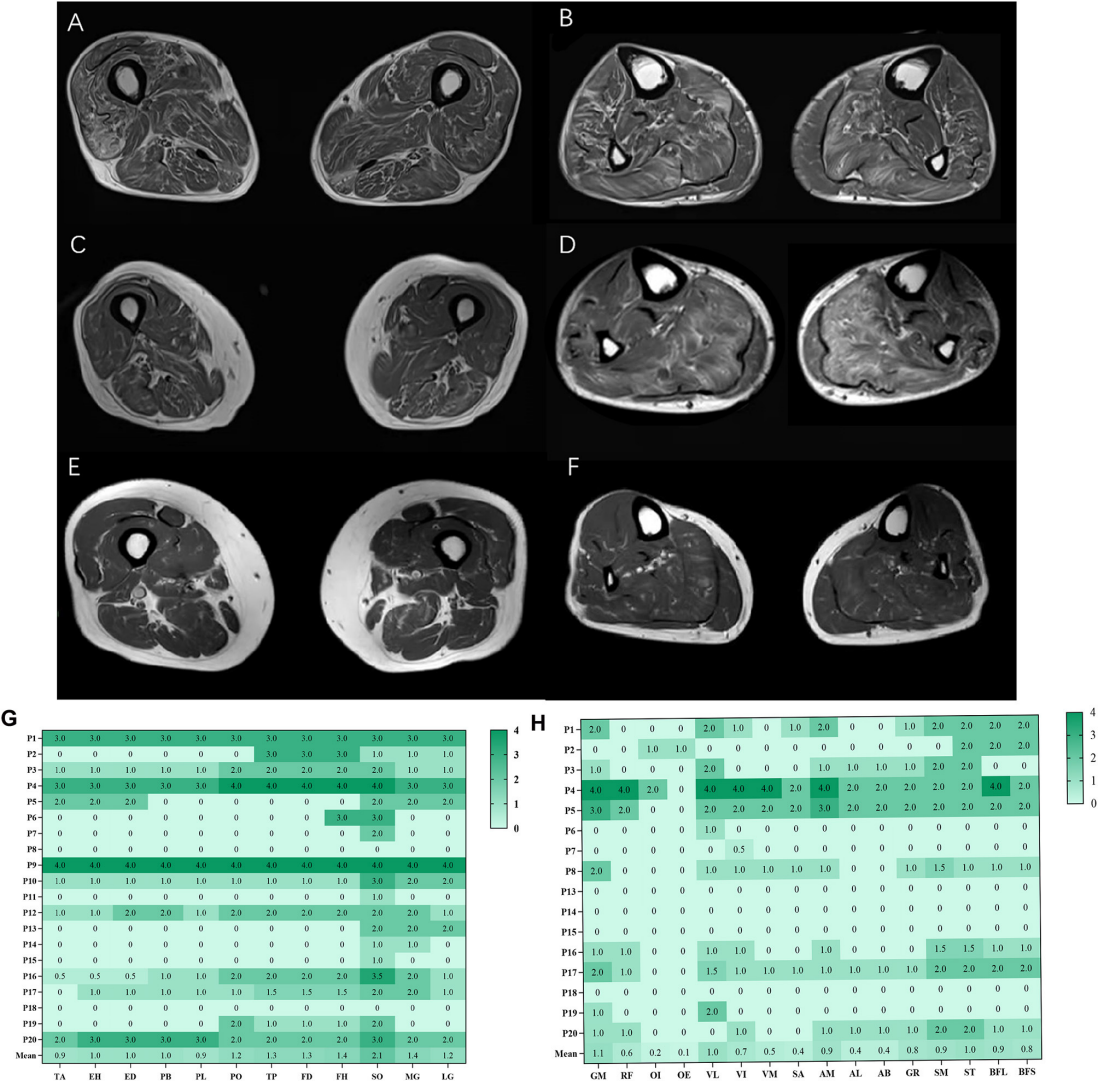
Fatty infiltration (bilateral mean values) at calf and thigh levels. Bilaterally symmetrical and diffused muscles on axial T1WI at the thigh level **(A)**, at the calf level **(B)**; the posterior compartments of muscles had severely fatty infiltration at the thigh level **(C)**, at the calf level **(D)**; patient 8 had more severe fatty infiltration of muscles at the thigh level **(E, F)**. The average score of fatty infiltration for each muscle was shown in heat maps **(G, H)**. GM, gluteus maximus; OI, obturator internus, OE, obturator externus; RF, rectus femoris; VL, vastus lateralis; VM, vastus medialis; VI, vastus intermedius; SA, Sartorius; AM, adductor magnus; AL, adductor longus; AB, adductor brevis; GR, gracilis; ST, semitendinosus; SM, semimembranosus, BFL, long head of biceps femoris; BFS, short head of biceps femoris; TA, tibialis anterior; EH, extensor hallucis longus, ED, extensor digitorum longus; PB, peroneus brevis; PL, peroneus longus; PO, popliteus; TP, tibialis posterior; FD, flexor digitorum longus; FH, flexor hallucis longus; SO, soleus; MG, medial head of gastrocnemius; LG, lateral head of gastrocnemius.

**Figure 2:**
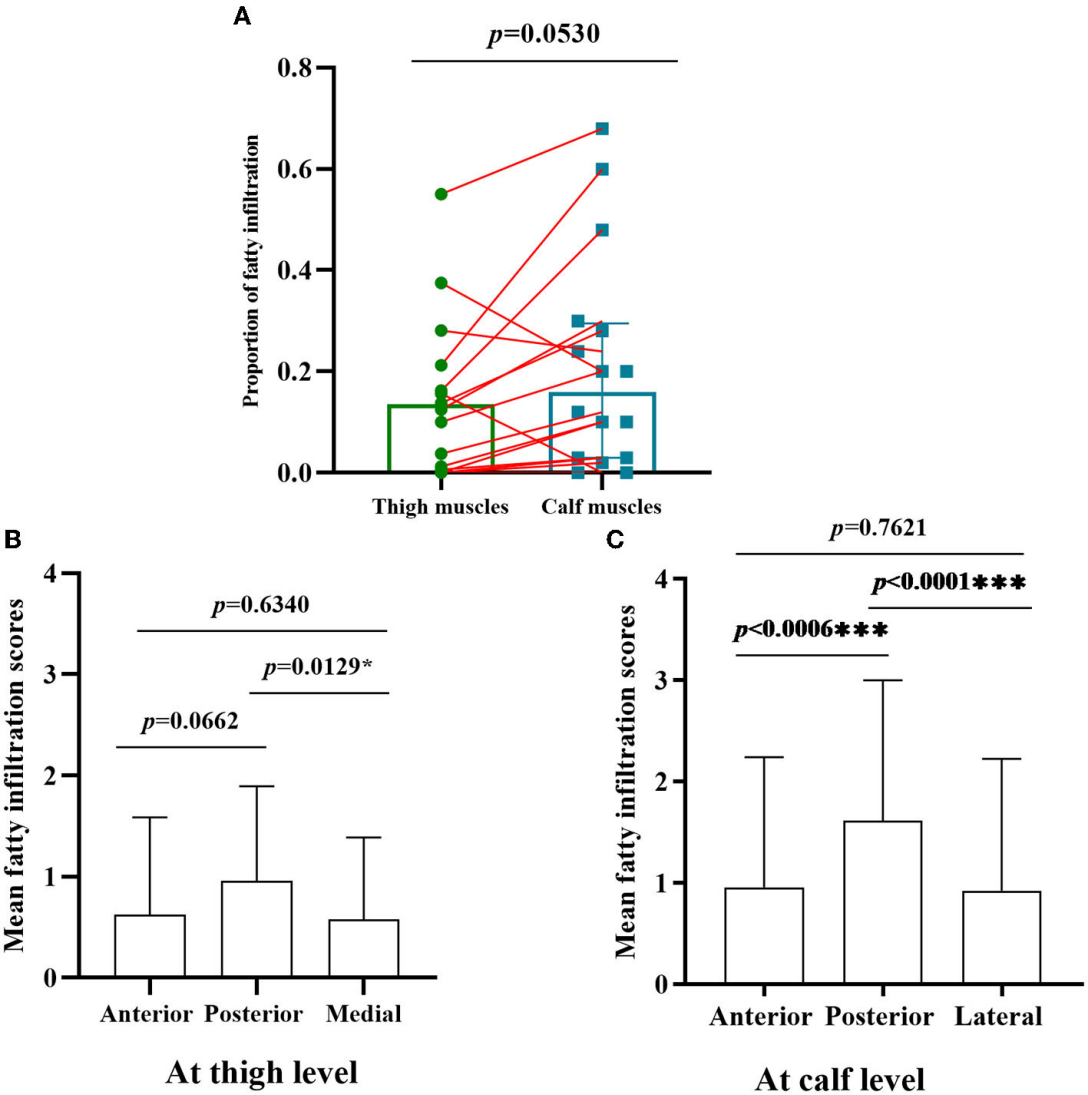
Comparison of fatty infiltration at calf and thigh levels, and different muscles compartments. **(A)** The proportion of fatty infiltration scores at the calf level was higher than that of the thigh level with paired comparison; **(B)** at the thigh level, the posterior compartments of muscles had severely fatty infiltration compared with medial compartments (*p* < 0.05); **(C)** the posterior compartments of muscles were heavily fatty at the calf level compared with anterior and lateral compartments *, ***Significant correlation at 0.05, 0.001 level, respectively. (all *p* < 0.001).

The overall distribution and extent of fatty infiltration of the involved muscles were bilaterally symmetrical and diffused on axial T1WI (Figures 1A,B). The average score of fatty infiltration for each muscle was shown in (Figures 1G,H). At the thigh level, the posterior compartments of muscles had more severely fatty infiltration than medial compartments (*p* < 0.05) (Figures 1C, 2B), and at the calf level, the posterior compartments of muscles were more heavily fatty than anterior and lateral compartments (all *p* < 0.001) (Figures 1D, 2C). The soleus muscle was the most heavily involved with higher fatty infiltration scores in all the patients except patient 2 (Figures 1D,H).

The proportion of fatty infiltration scores at the calf level was higher than at the thigh level with paired comparison (Figure 2A). However, there was no statistically significant difference (*p* = 0.0530) as three patients (patients 5, 8, and 17) had more severe fatty infiltration of muscles at the thigh level (Figures 1E,F). Patients 5 and 8 had no weakness on physical examination, and patient 17 had more severe weakness in proximal lower limbs than in distal ones.

**Figure 3:**
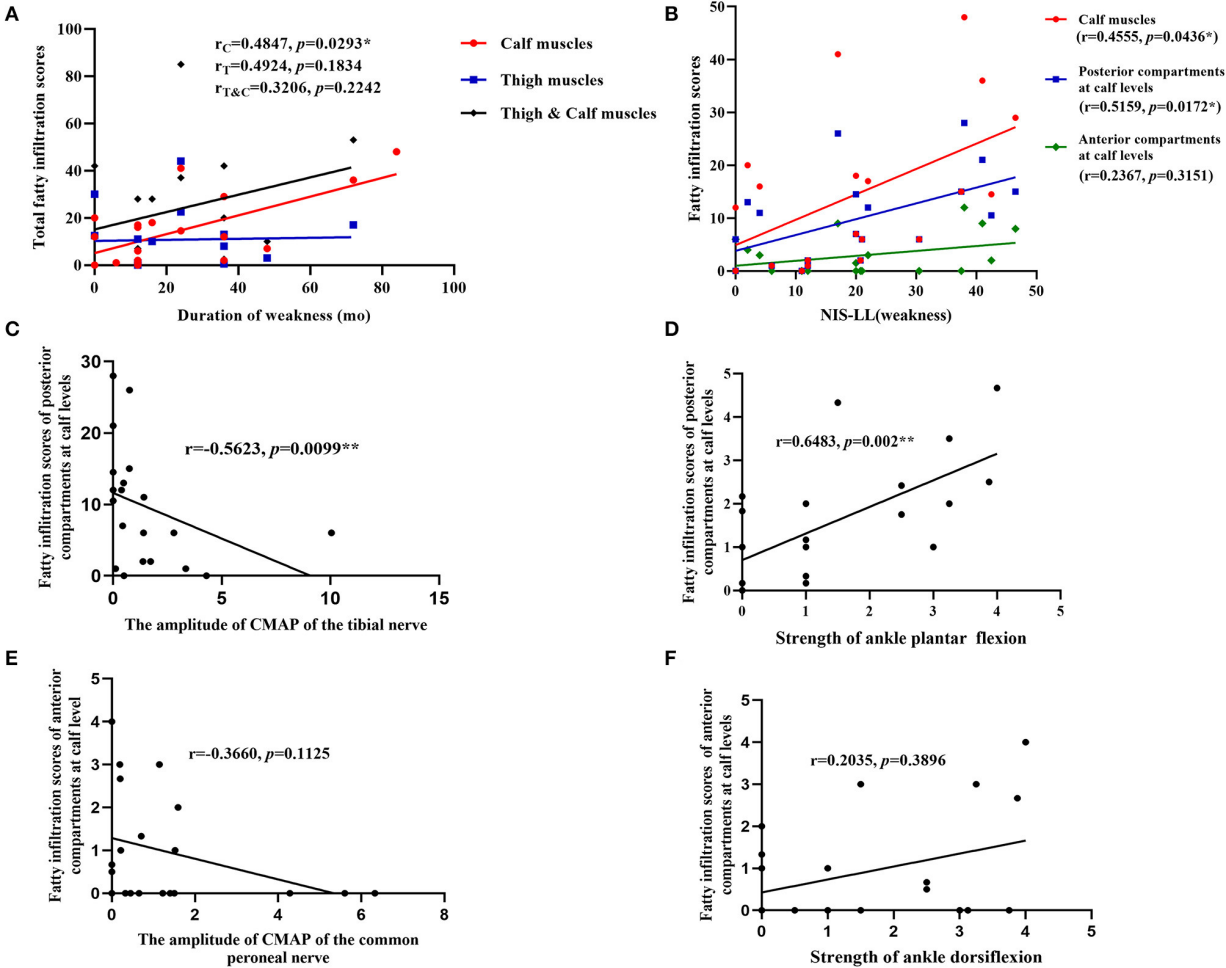
Correlations between parameters of muscle MRI and clinical data. **(A)** The fatty infiltration of calf muscles was positively correlated with the disease duration of weakness; **(B)** the fatty infiltration at the calf level and fatty infiltration of posterior compartments at the calf levels were positively correlated with NIS-LL (weakness); **(C)** the fatty infiltration of posterior compartments at the calf level was negatively correlated with the amplitude of the CMAP of tibial nerves; **(D)** the fatty infiltration of posterior compartments at the calf level was related to the strength of ankle plantar flexion muscles (NIS score); **(E)** the fatty infiltration scores of anterior compartments at the calf level was not related to the amplitude of the CMAP of common peroneal nerves; **(F)** the fatty infiltration of anterior compartments at the calf level was not related to the strength of ankle dorsiflexion muscles (NIS score). *, **Significant correlation at 0.05, 0.01 level, respectively.

Fatty infiltration scores of each patient were shown in Table 2. The correlations of fatty infiltration scores with clinical parameters were analyzed. The disease duration of weakness was positively correlated with fatty infiltration of calf muscles (*r* = 0.4874, *p* = 0.0293) (Figure 3A), but not with that of thigh muscles or total lower limbs. The fatty infiltration at the calf level was also positively correlated with NIS-LL (weakness) (*r* = 0.4555, *p* = 0.0436) (Figure 3B) and Fatty infiltration of posterior compartments at the calf level, where the most severe impairment of fatty infiltration occurred, was related to NIS-LL (weakness) (*r* = 0.5159, *p* = 0.0172) (Figure 3B), the amplitude of the CMAP of the tibial nerve (*r* = −0.5623, *p* = 0.0099) (Figure 3C), and the strength of ankle plantar flexion muscles (*r* = 0.6483, *p* = 0.002) (Figure 3D), but the fatty infiltration of anterior compartments at the calf level was not correlated with NIS-LL (weakness), (*r* = 0.2367, *p* = 0.3151) (Figure 3B) the amplitude of the CMAP of the common peroneal nerve (*r* = −0.3660, *p* = 0.1125) (Figure 3E), or the strength of ankle dorsiflexion muscles (*r* = 0.2035, *p* = 0.3896) (Figure 3F).

**Table 2:** Fatty infiltration scores of each patient.

	**Fatty infiltration of calf muscles**	**Fatty infiltration of thigh muscles**	**Fatty infiltration of total muscles^†^**	**Fatty infiltration of posterior compartments at calf levels**	**Fatty infiltration of anterior compartments at calf levels**
Patient 1	3.0 ± 0	1.1 ± 0.9	1.9 ± 1.2	3.0 ± 0	3.0 ± 0
Patient 2	1.3 ± 1.4	0.5 ± 0.8	0.8 ± 1.1	2.1 ± 1.1	0
Patient 3	1.4 ± 0.5	0.7 ± 0.8	1.0 ± 0.8	1.7 ± 0.5	1.0 ± 0
Patient 4	3.4 ± 0.5	2.8 ± 1.2	3.0 ± 1.0	3.7 ± 0.5	3.0 ± 0
Patient 5	1.0 ± 1.0	1.9 ± 0.8	1.5 ± 1.0	0.9 ± 1.1	2.0 ± 0
Patient 6	0.5 ± 1.2	0.1 ± 0.3	0.3 ± 0.8	0.9 ± 1.5	0
Patient 7	0.2 ± 0.6	0 ± 0.1	0.1 ± 0.4	0.3 ± 0.8	0
Patient 8	0	0.8 ± 0.6	0.4 ± 0.6	0	0
Patient 9	4.0 ± 0	NA	NA	4.0 ± 0	4.0 ± 0
Patient 10	1.3 ± 0.7	NA	NA	1.6 ± 0.8	1.0 ± 0
Patient 11	0.1 ± 0.3	NA	NA	0.1 ± 0.4	0
Patient 12	1.7 ± 0.5	NA	NA	1.9 ± 0.4	1.3 ± 0.6
Patient 13	0.5 ± 0.9	0	0.2 ± 0.6	0.9 ± 1.1	0
Patient 14	0.2 ± 0.4	0	0.1 ± 0.3	0.3 ± 0.5	0
Patient 15	0.1 ± 0.3	0	0 ± 0.2	0.1 ± 0.4	0
Patient 16	1.5 ± 0.9	0.6 ± 0.6	1.0 ± 0.8	2.1 ± 0.7	0.5 ± 0
Patient 17	1.2 ± 0.5	1.4 ± 1.0	1.3 ± 0.8	1.5 ± 0.4	0.7 ± 0.6
Patient 18	0	0	0	0	0
Patient 19	0.6 ± 0.8	0.2 ± 0.5	0.4 ± 0.7	1.0 ± 0.8	0
Patient 20	2.4 ± 0.5	0.8 ± 0.7	1.5 ± 1.0	2.1 ± 0.4	2.7 ± 0.6

*NA, not available*.

† *Only data on sixteen patients were available as four patients did not perform muscle MRI at thigh levels*.

*The fatty infiltration scores of individual muscles used a modified 0–5 Mercuri's point scale (15)*.

## Discussion

This study was intended to conduct an in-depth analysis of the clinical phenotypes and characteristics of MRI of muscles of lower limbs in 20 patients with ATTRv amyloidosis whose mean age was 44.2 years. In general, the distribution and extent of fatty infiltration of the involved muscles were symmetrical and diffused. Overall, the pattern of muscle fatty infiltration was consistent with the muscle weakness of the distal-to-proximal gradient, which pointed to secondary neurogenic muscle impairment that had been confirmed in other hereditary and acquired peripheral neuropathies (9, 10, 16–18).

Interestingly, there was selective muscle fatty infiltration in patients with ATTRv amyloidosis to some extent, the posterior compartments of muscles had a larger fat fraction in lower limbs, especially at the calf level, and the soleus muscle was the most heavily involved, which could be a helpful diagnostic marker, as was recently reported by Primiano et al. (19). Compared to the study by Primiano et al., it was found in our study that the fatty infiltration at the calf level was also positively correlated with NIS-LL (weakness). In Charcot-Marie-Tooth (CMT) 1A, the largest fat infiltration was quantified in anterior and lateral compartments at the calf level, and selective intrinsic foot muscles involvement was typical of CMT 1A cases with minimal disease signs (16, 20). In CMT 2 cases, massive fatty atrophy of foot musculature was observed in muscle MRI (21). The distal-to-proximal gradient was also present in hereditary neuropathy with liability to pressure palsies, which was not a length-dependent neuropathy clinically (10). For acquired peripheral neuropathies, both anterior and posterior leg compartments were affected as in chronic inflammatory demyelinating polyneuropathy (CIDP) (18). In diabetic polyneuropathy, atrophy was the most pronounced in distal muscles of the lower leg, indicating a length-dependent neuropathic process, while abnormalities were the most pronounced in the plantar flexors by MRI assessment of skeletal muscles (17, 22). Limb-girdle muscle fatty infiltration in MRI could help monitor the evolution of amyotrophic lateral sclerosis (23). Good knowledge of denervation patterns by muscle MRI would facilitate the determination of the nerves involved and the extent of nerve entrapment (24). The relatively selective soleus muscle involvement might be a specific indicator of ATTRv amyloidosis, and posterior compartments seemed to confirm a predominant denervation pattern of tibial nerves.

However, three of these patients had more severe proximal fatty infiltration in the muscle MRI, with no or predominant proximal weakness. A previous study in the UK also described the proximal weakness in some patients (25). It was found that amyloid deposition throughout nerves might help account for proximal weakness (26). Furthermore, proximal weakness in lower limbs was also present in other polyneuropathies, such as CMT and CIDP (9, 27). However, previous studies also reported primary skeletal muscle impairments relating to amyloid deposits in ATTRv amyloidosis cases (6–8), which was why the involvement of myogenic injury should not be overlooked. Muscle fatty infiltration could occur before clinical weakness appeared, especially in proximal muscle, which demonstrated that the muscle MRI might be sensitive to motor dysfunction in ATTRv amyloidosis. The fatty infiltration of clinically normal muscles in CMT1A was also reported (20).

According to our study, the fatty infiltration at the calf level, especially in the posterior compartments, was quite consistent with NIS-LL (weakness). Fatty infiltration of posterior compartments at the calf level could reflect the strength level of ankle plantar flexion muscles and amplitude of the CMAP of tibial nerves, but that of anterior compartments could not reflect the strength level of ankle dorsiflexion muscles or the amplitude of the CMAP of common peroneal nerves, suggesting that the clinical and electrophysiological manifestations may not match the imaging feature perfectly to some extent, and there may be different compensatory mechanisms after muscle injury of anterior and posterior compartments.

In conclusion, we found that the pattern of muscle fatty infiltration was consistent with a distal-to-proximal gradient on the whole in ATTRv amyloidosis. There was an inverse gradient of fatty infiltration in some patients, and the proximal weakness of lower limbs could also occur in ATTRv amyloidosis. There was selective fatty infiltration of posterior compartments of muscles in patients with ATTRv amyloidosis to some extent and fatty infiltration of the soleus muscle might be a marker of progression of ATTRv amyloidosis.

## Data Availability Statement

The original contributions presented in the study are included in the article/supplementary material, further inquiries can be directed to the corresponding authors.

## Ethics Statement

The studies involving human participants were reviewed and approved by the Ethic Committee of Peking University First Hospital. The patients/participants provided their written informed consent to participate in this study.

## Author Contributions

XC: acquisition of data, completion of statistical analysis, drafting of the initial manuscript, and writing of the final manuscript. KD: acquisition of data, study concept and design, completion of statistical analysis, and critical revision of the manuscript. YT, XZ, MY, YZ, JD, HL, and WZ: study concept and design, and critical revision of the manuscript. YY and LM: data review, interpretation of results, and revision of the initial draft. All authors contributed to the article and approved the submitted version.

## Funding

This study was supported by the Beijing Municipal Natural Science Foundation (No. 7194323).

## Conflict of Interest

The authors declare that the research was conducted in the absence of any commercial or financial relationships that could be construed as a potential conflict of interest.

## Publisher's Note

All claims expressed in this article are solely those of the authors and do not necessarily represent those of their affiliated organizations, or those of the publisher, the editors and the reviewers. Any product that may be evaluated in this article, or claim that may be made by its manufacturer, is not guaranteed or endorsed by the publisher.
